# ReCGiP, a database of reproduction candidate genes in pigs based on bibliomics

**DOI:** 10.1186/1477-7827-8-96

**Published:** 2010-08-14

**Authors:** Lun Yang, Xiangzhe Zhang, Jian Chen, Qishan Wang, Lishan Wang, Yue Jiang, Yuchun Pan

**Affiliations:** 1 School of Agriculture and Biology, Shanghai Jiao Tong University, Shanghai, 200240, China; 2 Bio-X Center, Key Laboratory for the Genetics of Developmental and Neuropsychiatric Disorders (Ministry of Education), Shanghai Jiao Tong University, Shanghai, 200240, China; 3 Shanghai Key Laboratory of Veterinary Biotechnology, Shanghai, 200240, China

## Abstract

**Background:**

Reproduction in pigs is one of the most economically important traits. To improve the reproductive performances, numerous studies have focused on the identification of candidate genes. However, it is hard for one to read all literatures thoroughly to get information. So we have developed a database providing candidate genes for reproductive researches in pig by mining and processing existing biological literatures in human and pigs, named as ReCGiP.

**Description:**

Based on text-mining and comparative genomics, ReCGiP presents diverse information of reproduction-relevant genes in human and pig. The genes were sorted by the degree of relevance with the reproduction topics and were visualized in a gene's co-occurrence network where two genes were connected if they were co-cited in a PubMed abstract. The 'hub' genes which had more 'neighbors' were thought to be have more important functions and could be identified by the user in their web browser. In addition, ReCGiP provided integrated GO annotation, OMIM and biological pathway information collected from the Internet. Both pig and human gene information can be found in the database, which is now available.

**Conclusions:**

ReCGiP is a unique database providing information on reproduction related genes for pig. It can be used in the area of the molecular genetics, the genetic linkage map, and the breeding of the pig and other livestock. Moreover, it can be used as a reference for human reproduction research.

## Background

Pork is the major red meat source worldwide, which contributes to forty-three percent of the world's red meat consumed [1]. Good pork production requires that pigs have high levels of reproduction, meat quality, carcass merit, disease resistance, and survivability [1]. The improvement of reproductive performance in pigs has attracted great attentions of researchers, because moderate increases in litter size will lead to great economic benefits [2]. However, traditional selection methods based on estimated breeding value are expensive, laborious and time consuming. Moreover, it results in only low genetic gain. This is why it is important to understand the genetic basis of traits affecting reproduction and to use the marker assisted selection method (MAS) in order to achieve more straightforward results. At present, there are two methods to identify genetic markers: the genome scans approaches and the candidate gene approach. The candidate gene approach is the most direct method of testing association between a gene and a phenotype [3] and can be utilized in any populations with a well-known pedigree in which phenotypes can be measured [4]. The candidate gene approach has been proved extremely powerful for studying the genetic architecture of reproduction traits. For example, with this approach, Rothschild et al. [5] demonstrated that there was a significant correlation between the estrogen receptor gene (*ESR*) and litter size.

In principle, candidate gene approach can be applied if a gene has a potential influence on a phenotypical trait (physiological candidate), located in a narrowed QTL region (positional candidate) or has an influence on the phenotypical trait in other species (comparative candidate) [6]. However, there were limited positional candidates related to pig's reproduction traits so far. So, candidate genes for reproduction traits in pig are mainly physiological or comparative candidate.

Most of researchers choose candidate genes by extensive literature reading. Electronic literature is now growing rapidly in companion with the development of the life science. At the same time, more and more databases appear. The database of PubMed biomedical literature has over 19,470,000 entries and embraces almost every field of life sciences. Every year, over 600,000 literatures are published. It is not feasible for a researcher of a particular area to read all the papers in his field, let alone the literature in the related field. As about the pig, we still don't get the full map of its genome and our knowledge about reproductive candidate genes is limited. The databases for genome, karyotype, genome mapping, EST, SNP and QTL of pig have been established [7], however, there is a webpage listing catalog of porcine genes of interest in endocrinology and reproduction [8]. There is still no database bearing up-to-date candidate genes for reproduction traits of pig. Based on genetic similarity between human and pig and the intensive studies on human reproductive genetic mechanism, we developed ReCGiP (Database of Reproduction Candidate Genes in Pigs based on bibliomics).

According to comparative genomics studies, human candidate reproduction genes can be used as reference counterpart of that in pigs. By mining and analyzing the biomedical literature database using natural language processing technology, we builded the ReCGiP, which provides candidate genes related to six main reproductive processes including spermatogenesis, oogenesis, fertilization, preimplantation development, embryo implantation and placental development. Other genes related information, such as associated literatures, KEGG pathway, GO annotation and OMIM information. The gene-gene co-occurrence networks [9-11] were also included where a line was drawn between two genes if two genes were co-cited in one Pubmed abstract. ReCGiP provides genes which are associated with the reproductive processes and the reproductive traits, and related literature information. The database will facilitate the researchers to choose their interesting genes for the experimental design.

## Methods

### Construction of pig reproduction related bibliome

In order to show the current DNA level research on pig reproductive processes and reproductive traits, we constructed porcine reproduction related literature group. This literature group contains seven themes, of which six are the main reproductive processes (spermatogenesis, oogenesis, fertilization, preimplantation development, embryo implantation and placental development), one is the reproductive trait. Sixty-three key words of seven themes were chosen from MeSH of NCBI, PigQTLdb [12-14] and by our biology knowledge. Pig related keywords contained pig, porcine, swine, boar, piglet, gilt, "*Sus scrofa*" and their plural form. Pig related PubMed entry records in the mentioned seven themes stored in MEDLINE form were retrieved by searching PubMed using eSearch and eFetch with SQL statement which was constructed by the selected search terms and boolean logic operators. In order to obtain gene name in the literatures, we analyzed MEDLINE format files using the gene/protein names recognition software AbGene [15], and checked the result manually. The correct gene names, their gene ID and the relevant literature fell into corresponding categories. All the data were deposited into the rational database MySQL 5.0.0.

### Construction of comparative genomics bibliome

Based on comparative genomics, we constructed the comparative genomics bibliome by mining articles about the same six themes of human genes. Firstly, 40 key words were chosen from MeSH of NCBI and by our biology knowledge. These words included spermatogenesis, oogenesis, fertilization, acrosome reaction, preimplantation embryo, embryo implantation, decidualization, placenta, and so on. Records were retrieved by searching PubMed using eSearch and eFetch with SQL statement consisted of search terms and boolean logic operators. PubMed entries in the form of XML were deposited into the rational database MySQL 5.0.0. The reference impact factor for each PubMed entry is the mean impact factor of the relevant journal in the science citation index from 2003 to 2007. The gene-pubmed index was downloaded from the FTP site of Entrez Gene [16]. PubMed entries with indexed genes more than five were dismissed due to absence of specificity in the gene-pubmed relationships.

### Construction of the gene-gene co-occurrence network based on comparative genomics

If two genes co-occurred in a PubMed entry, they tend to relate with each other biologically directly or indirectly [11,17,18]. So a biological relationship will be established between the two genes if they are co-cited in at least two PubMed entries. The gene-gene co-occurrence networks (GGCON) of the six themes mentioned above were constructed based on the genes' co-citation, and were changed into XGMML format [19] so as to be visualized in the jSquid [20].

### Assignment of the core and the extended genes

Genes in the reproduction related theme 'T' were retrievable from the gene-pubmed index provided by GeneRIF [21] of Entrez Gene. For gene i, the values *a*_*i *_and *b*_*i*_, represented the number of co-occurrence with theme T related PubMed entry and non-theme T related PubMed entry respectively, while *c*_*i *_and *di *represented number of non co-occurrence with theme T related PubMed entry and non-theme T related PubMed entry respectively. The co-occurrence ratio (*CR*), which denotes the ratio between the citation rate of this gene in theme-T and the citation rate of this gene in all Pubmed entries, was calculated as:

(1) $$CR_{i}=\left(\frac{a_{i}}{a_{i}+c_{i}}\right)\left(\frac{a_{i}+c_{i}+b_{i}+d_{i}}{a_{i}+b_{i}}\right)$$

A gene was defined as a "core" gene if its *CR *exceeded zero with *a *and *c *above three. Genes, with *CR *equal to zero but connected to "core" genes in the GGCON, were assigned to extended genes. Genes, of which the *CR *were greater than zero and with either *a *or *c *less than three were also assigned to extended genes, with their *CR *being set to zero. For T, the core and extended genes can be regarded as genes which were directly or indirectly associated with T, and the *CR *value of them reflected their relevance in the theme T.

### Enrichment of reproduction associated pathways

Core and extended genes with *CR *value greater than 0.001 were included in the KEGG orthology enrichment analysis [22] using KOBAS [23]. The enriched terms and the contributing genes were deposited in our database.

### Database implementation

ReCGiP database run on linux system environment using MySQL (version 5.0.0) as the database engine. We developed a data integrate pipeline to incorporate PubMed, GO annotation, KEGG pathway and OMIM. All data integrate pipeline are developed using standard Perl modules, such as BioPerl, DBI, and POD. Standard Perl modules DBI and CGI were used in the Web Interface design.

## Results and Discussion

### Database contents

#### Pig gene

This part contains genes under seven themes including reproductive processes (spermatogenesis, oogenesis, fertilization, preimplantation development, embryo implantation and placental development) and reproductive traits (litter size, ovulation rate, teat number and so on). This part is composed of two kinds of pages: theme gene page and gene page.

Theme gene page includes all the relevant genes of one theme sorted by the number of articles which could prove that gene is related to this theme. Each page shows 10 genes. Each gene has a hyperlink to Entrez Gene, and at least one article as evidence. Each literature has hyperlinks to PubMed. Gene page provides detail information of the literatures including subject title, author, abstract, journal name, published date, impact factor and other information.

Four genes (*ESR1*, *PRLR*, *RBP4 *and *FSHβ*), which were identified to be related significantly to the litter traits in different breeds and populations [6], are included in topics for the reproductive traits of the ReCGiP database. These four genes are listed in sequence of *ESR1*, *FSHβ*, *PRLR *and *RBP4*, according to the amount of related literatures. Two of the genes *ESR1 *and *FSHβ *have been applied in production. China Agricultural University cooperating with many domestic pig breeding companies has improved the litter size 0.5-1.5 by using *FSHβ *gene in breeding.

We provide pig candidate gene sorted by their association with reproductive traits. Researchers can choose gene of their interest based on these information to design the experiment in order to understand the genetic mechanism of porcine reproductive-related traits.

#### Comparative genomics

**Figure 1:**
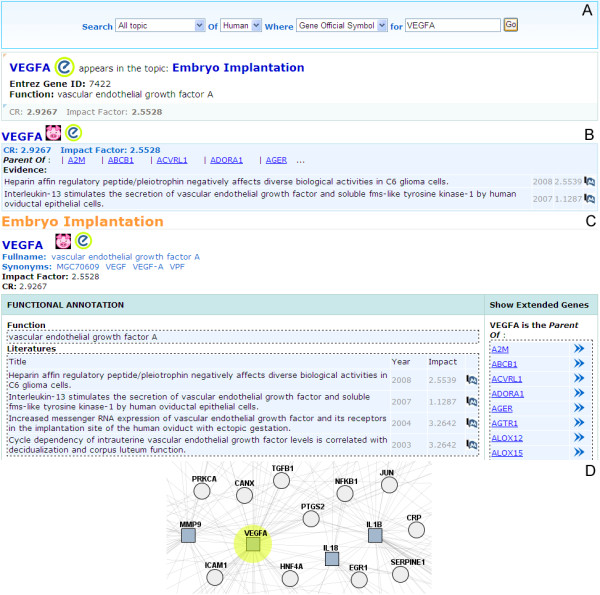
**The search interface and various features of ReCGiP**. (A) search for association topics of a given gene, (B) theme gene page, (C) gene page and (D) gene-gene co-occurrence network.

This part includes six themes (spermatogenesis, oogenesis, fertilization, preimplantation development, embryo implantation and placentation) of human genes. This part is also composed of two kinds of pages: theme gene page and gene page. Each theme gene page includes the relevant genes sorted by *CR *values with 10 genes showed in per page (see Figure 1B). *CR *value indicates correlation between gene and theme.

Gene page consists of two parts: (1) functional annotation, (2) core or extended gene. Each gene page has a separate web site and the external databases can be linked directly to the page. The functional annotation section provides functional gene annotations and gene biomedical literatures as evidence obtained from NCBI [24], GO [25], KEGG [22] and OMIM [24] (see Figure 1C). Core gene(s) or extended gene(s) section provides the co-occurrence gene list relevant to this gene on the right of the page (see Figure 1C). At present, 3155 human genes including core and extended genes are available in our database. Eight hundred and sixty-one homologous genes were defined for pig by gene nomenclature [26,27]. The summarization of key data of ReCGiP was listed in Table 1.

**Table 1:** Information of reproductive candidate genes

Topic	Gene count	PubMed	GO	KEGG pathway	The corresponding pig gene count
**Core gene**					
spermatogenesis	142	533	467	56	50
oogenesis	36	95	238	36	21
fertilization	105	329	505	73	57
preimplantation	7	21	49	8	4
embryo implantation	64	224	378	63	34
placentation	41	101	277	55	20
**Extended gene**					
spermatogenesis	1480	175	2462	144	448
oogenesis	1015	68	2069	153	376
fertilization	1973	140	2824	173	607
preimplantation	211	11	779	106	73
embryo implantation	2057	112	2836	163	600
placentation	1564	69	2521	162	499

#### Network

We constructed six gene co-occurrence networks on themes of spermatogenesis, oogenesis, fertilization, preimplantation development, embryo implantation and placental development based on NCBI's PubMed literature database. In a particular theme, we can infer that genes in the hub of the co-occurrence network may play an key role in the selected theme and may be the preferred candidate gene for experiment researches. Since network diagram of each subject is too large to display on a static page, co-occurrence networks are visualized by an interactive Java plug-in. We will take "embryo implantation" theme as example to show the functions and the applications of the co-occurrence network.

We may search gene, such as gene *VEGFA*, in gene co-occurrence network. When you enter the "VEGFA" in the search box at lower-left corner, *VEGFA *gene as a node of co-occurrence in its network is highlighted (see Figure 1D). The blue squares indicate core genes and the circles represent extended genes. Zoom-out view can be obtained by sliding a zoom slider at the bottom panel. If you select node VEGFA under the theme of embryo implantation, gene nodes, which share the same network with VEGFA, and their edge with VEGFA will be highlighted. From gene co-occurrence network, you can see that the *VEGFA *gene is at core position, associated with the 236 genes, including five genes of MMPs family (*MMP1*, *MMP3*, *MMP7*, *MMP11*, and *MMP14*). There have been a large number of studies which showed that family members of MMPs play important roles in the reproductive process [28,29]. A study in our laboratory focused on relationship between *MMP3 *gene polymorphisms and pig reproductive traits found that *DraI-RFLP *polymorphic loci of *MMP3 *gene had significant effect on the EBV (Estimated Breeding Value). When you select VEGFA node and then click the right mouse button, the menu bar of the VEGFA gene-page hyperlinks will appear. We can get the gene function information, relevant literatures, KEGG pathways and GO annotations and other relevant information through the link.

#### Pathway

Biological pathway can help us to understand function of cells and organisms. Some signaling pathways are conservative between organisms with distant genetic relationship[30]. Understanding these pathways in different organisms can help us to understand signal transduction mechanisms and their evolutionary relationship. The ReCGiP database provides pathways enriched by reproduction-related candidate genes in spermatogenesis, oogenesis, fertilization, preimplantation development, embryo implantation and placental development. If genes of a subject were enriched at several signaling pathways or cancer pathways, the genes of the pathway may be related closely to that topic and thus affect the reproductive process.

### Utility of the database

There are various ways by which users can access data stored in ReCGiP. The database can be queried by gene symbols and Entrez Gene ID to get information about genes, KEGG pathway, GO annotation, and extracted evidences from existing PubMed biomedical literature. In addition, ReCGiP can be browsed by selecting a specific species from the home or content pages. Figure 1 shows the search interface and the result. ReCGiP is publicly accessible at [31].

### Examples for using ReCGiP

Example 1: how to obtain Subject-related genes (take "embryo implantation" theme as example)

Choose "comparative genomics" on the navigation bar, and choose "embryo implantation", then 64 core genes of the selected subject ranked by *CR *values from high to low will be shown. The gene with the highest *CR *is *BYSL*. BYSL protein was first found by Fukuda through the yeast two-hybrid assay [32]. It is a cell adhesion molecule, plays an important role in embryo implantation and ribosome biosynthesis [33].

Example 2: What topics are related to a given gene or if a gene is associated with a particular topic (take gene *VEGFA *as example)

Select all themes query, use "human *VEGFA *gene" or its Entrez gene ID as the keyword, you will find that the gene is relevant to five themes. The theme which is not related to this gene is preimplantation development. The number of studies about human embryonic preimplantation development is scarce due to ethical reasons. Few genes related with preimplantation genetic development were obtained in our database and this could be why *VEGFA *was not lead to preimplantation development. The detailed information of this query is shown in Figure 1C. From the result, we can find that *VEGFA *is a core gene in the four topics and a extended gene in one topic (see Figure 1A). The 236 extended genes in embryo implantation theme are expanded by the *VEGFA*, such as *A2M*, *ABCB1 *and so on. *VEGFA *is the most important regulator of blood vessel formation and plays an important role in embryo implantation and early pregnancy blood vessel changes [34-36]. Therefore, we can predict that *VEGFA *is essential in spermatogenesis, oogenesis, fertilization, preimplantation development, embryo implantation and placental development and thus *VEGFA *could serve as an important candidate gene for research of porcine reproductive traits.

### Future developments

We will divide up the reproductive traits theme into their different traits: litter size, ovulation rate, teat number, etc, which are some of the most interested traits for breeders. Future research also includes enriching the database by adding information on mouse/rat reproduction genes as references for candidate genes of pig reproduction traits. In addition, machine learning or other NLP techniques will be applied to compare the results with the supervised study. Our long-term goal is to make ReCGiP part of the major information resources integrating PubMed, GO terms and KEGG pathways for pigs and for other livestock.

## Conclusions

The ReCGiP database aims to provide a resource of candidate genes for pig reproduction. ReCGiP currently includes 3155 human genes and 861 pig genes. In addition, it contains 179 KEGG pathways, 3507 GO annotations, 1706 references, enrichment pathways of these genes and gene-gene co-occurrence networks under six reproduction topics. We also give the significance of genes in accordance with the number of co-occurred genes in gene-gene co-occurrence network. In addition, we have sorted core genes by *CR *value, so it is easy to make sure the importance of each gene under different themes. The ReCGiP server is the first specialized database on pig candidate genes which provides information of possibly reproductive traits associated genes. Pig genome is not fully released yet and there are still many hypothetical and unknown genes, but ReCGiP provides a platform for biologists to annotate them by experimental verification and instruct breeding work. Moreover, it can be used as a reference for human reproductive research.

## Abbreviations

GO: Gene Ontology; KEGG: Kyoto Encyclopedia of Genes and Genomes; NCBI: National Center for Biotechnology Information; OMIM: Online Mendelian Inheritance in Man; PERL: practical extraction and report language; PERL CGI: Common gateway interface scripting with perl; PERL DBI: Database interface (Standard database interface module for perl); PHP: PHP hypertext pre-processor; SQL: structured query language; XML: extensible markup language.

## Competing interests

The authors declare that they have no competing interests.

## Authors' contributions

YCP and XZZ had the initial ideas for this study. LY, XZZ and YCP designed the concept of the methods and the database schema, and drafted the manuscript. LY, XZZ, JC and QSW wrote the code, designed the web site and set up the local databases. The example screening dataset was provided by XZZ, YJ and LSW. YCP and LSW revised the manuscript. All authors read and approved the final manuscript.
